# Draft whole genome of *Bacillus velezensis* strain QA2 rhizobacterium isolated from quinoa (*Chenopodium quinoa* Willd.)

**DOI:** 10.1128/mra.00973-25

**Published:** 2025-11-12

**Authors:** Abdussabur M. Kaleh, Oumaima Ezzahidi, Ismail Mahdi, Abdelmounaaim Allaoui, Nidal Fahsi, Latefa Biskri, Joann K. Whalen

**Affiliations:** 1 Center for Sustainable Soil Science (C3S), Mohammed VI Polytechnic University 479571, Ben Guerir, Morocco; 2 African Genome Center, Mohammed VI Polytechnic University 479571, Ben Guerir, Morocco; 3 Microbiology Laboratory, Mohammed VI Polytechnic University 479571, Ben Guerir, Morocco; 4 Department of Natural Resource Sciences, McGill University 444590, Montreal, Québec, Canada; University of Maryland School of Medicine, Baltimore, Maryland, USA

**Keywords:** *Bacillus*, genome analysis, DNA sequencing

## Abstract

We sequenced the draft whole genome of *Bacillus velezensis* strain QA2 isolated from the rhizosphere soil of quinoa in Morocco to identify genes associated with potential plant growth-promoting and stress tolerance traits. The annotated genome offers insight into its genetic potential for agricultural applications.

## ANNOUNCEMENT

The bacterium *Bacillus velezensis* QA2 was selected for whole-genome sequencing (WGS) due to its potential as a plant growth-promoting bacterium with phosphate-solubilizing and abiotic stress-tolerant traits (1). Understanding the genetic basis of these functions is important in developing it as a bioinoculant for agriculture. QA2 was isolated from the rhizosphere soil of 3-month-old quinoa (*Chenopodium quinoa*) plants growing at the Agricultural Innovation and Technology Transfer Center of Mohammed VI Polytechnic University, Ben Guerir, Morocco (32^o^13′11″ N, 7^o^53′32″ W). Quinoa roots were collected by digging with sterile tools at the ripening stage in June. Soil tightly adhering to roots was collected and stored at 4°C for microbial isolation. One gram of soil was serially diluted and plated on trypticase soy agar (VWR, Casablanca, Morocco) and NBRIP agar plates (National Botanical Research Institute’s phosphate) (glucose 10 g, Ca_3_(PO_4_)_2_ 5 g, MgCl_2_·_6_H_2_O 5 g, MgSO_4_·7H_2_O 0.25 g, KCl 0.2 g, and (NH_4_)_2_SO_4_ 0.1 g) to distinguish phosphate-solubilizing bacteria. Isolates forming colonies with clear halo zones were selected and stored in 10% DMSO at −80°C. From 79 isolates, QA2 was distinguished by its high phosphate solubilization potential, tolerance to salinity (up to 14% NaCl), and high temperatures (up to 45°C) (1).

Genomic DNA was extracted from cultures grown in Luria Bertani broth at 28°C for 24 h with agitation (150 rpm) using the *DNeasy UltraClean Microbial Kit* (Qiagen, Hilden, Germany). DNA quality and quantity were assessed using Qubit (Thermofisher Scientific, USA). WGS libraries were prepared from 1 ng of DNA using the Nextera DNA library prep kit (Illumina, USA). The libraries were sequenced on an Illumina NextSeq 500/550 platform (AGC genomic Lab UM6P, Morocco) with a 150 bp paired-end configuration according to the protocol described by reference 2. Raw reads quality was assessed using FastQC and trimmed with Trimmomatic version 0.39 (3) to remove low-quality and adapter sequences (Phred score < 20). The quality-filtered reads were then assembled *de novo* using SPAdes (v3.12.0) (4). SPAdes was run in both standard and “careful” modes to maximize assembly accuracy. Assembly quality was evaluated with QUAST (v5.0.2) (5), and genome completeness was evaluated using BUSCO v5.3.1 (Benchmarking Universal Single-Copy Orthologs) (6). Genome annotation was conducted with Prokka v1.14.5 (7, 8), predicting 3,892 protein-coding genes, 6 rRNAs, 101 tRNAs, and 1 mRNA. Relevant statistics for the assembly and raw reads are listed in Table 1. All software was run with default settings.

**TABLE 1:** The genome of *Bacillus velezensis* QA2: summary statistics

Parameter		Data	
Raw genomic sequencing reads			
GenBank accession		NZ_CP194229.1	
No. of reads		1,848,258	
Total length (bp)		277,238,700	
*N*_50_ (bp)		107,547	
Genome sequence			
No. of sequences (contigs)		4	
Length (bp)		4,018,828	
GC content (%)		46	
Genome coverage (×)		≈69.2	
Gene annotation			
Total no. of genes		4,000 features (genes)	
No. of protein-coding genes		3,892	
No. of rRNAs		6 rRNA	
No. of tRNAs		101 tRNA	
No. of tmRNAs		1 mRNA	

BUSCO analysis			
Results (%)	Count (*n*)		Percentage (%)
Complete (C)	449		99.8
Single-copy (S)	447		99.3
Duplicated (D)	2		0.4
Fragmented (F)	1		0.2
Missing (M)	0		0.0
Total BUSCO groups searched	450		100.0

## Data Availability

The genome assembly of *Bacillus velezensis* QA2 has been deposited under the NCBI GenBank accession number (NZ_CP194229.1), BioProject ID (PRJNA1267493), and BioSample (SAMN48721967), respectively.
